# Comparing the International Clinical Diabetic Retinopathy (ICDR) severity scale

**Published:** 2023-07-07

**Authors:** Charles Cleland

**Affiliations:** 1 Clinical Research Fellow: International Centre for Eye Health, London School of Hygiene & Tropical Medicine. London, UK.

**Table:** 

ICDR severity scale	Scottish grading protocol	English protocol	Example retinal images with labelled features
**Retinopathy grade**			
**No diabetic retinopathy (DR)**	**R0**	**No DR (R0)**	**Normal retina**
**Mild non-proliferative diabetic retinopathy (NPDR)** Microaneurysms only	**R1** Microaneurysms or haemorrhages with or without hard exudates	Background DR (**R1**)	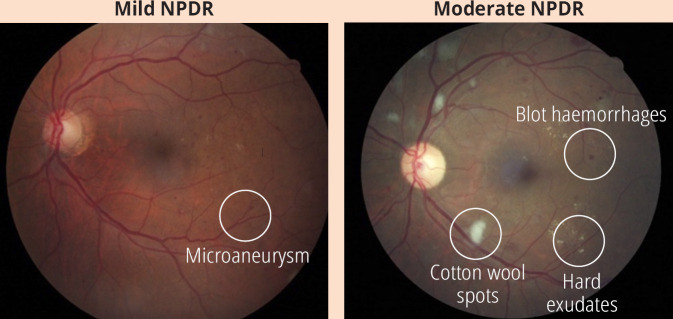
**Moderate NPDR** Any of the following: microaneurysms retinal dot or blot haemorrhages hard exudates or cotton wool spots No signs of severe NPDR	**R2** 4 or more blot haemorrhages in 1 hemifield only		
**Severe NPDR** More than twenty intraretinal haemorrhages in all 4 quadrants Definite venous beading in 2 or more quadrants Prominent intraretinal microvascular abnormality (IRMA) in 1 or more quadrants No signs of proliferative retinopathy	**R3** 4 or more blot haemorrhages in superior and inferior hemifields	Pre- proliferative DR (**R2**)	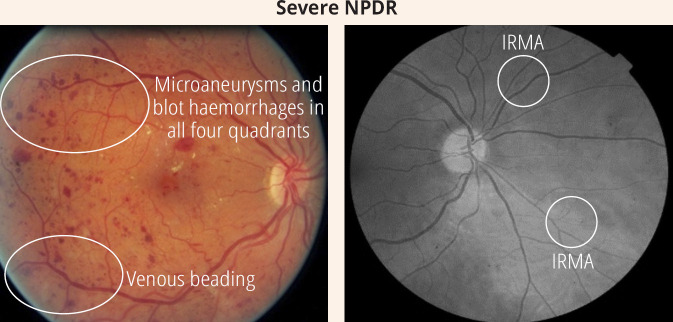
**Proliferative DR** One or both of the following: neovascularisation (new vessels) vitreous/pre-retinal haemorrhage	**R4**	Proliferative DR (**R3**)	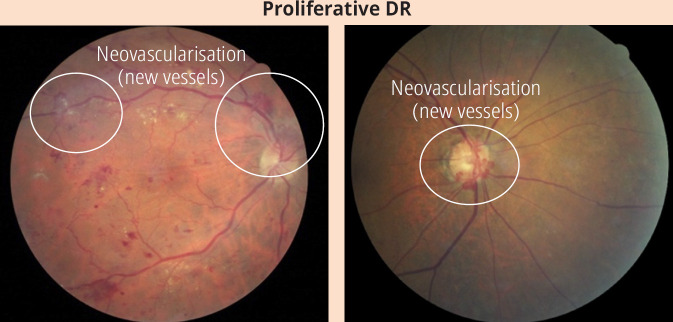
**Maculopathy grade**			
**No macular oedema** No exudates or apparent retinal thickening within one disc diameter (DD) of the fovea	**M0**	**M0**	**Normal macula**
**Macular oedema** Exudates or apparent thickening within 1 DD of the fovea	**M1** Any lesions within 2 DD but more than 1 DD from the fovea	**M1**	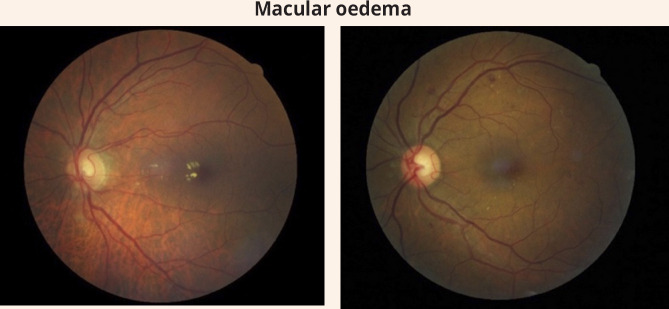 Examples of maculopathy showing exudates within 1 disc diameter (DD) of the fovea
	**M2** Any lesions < 1 DD from the fovea		

DR: diabetic retinopathy, NPDR: non-proliferative diabetic retinopathy, IRMA: intraretinal microvascular abnormality, DD: disc diameter

